# Clinical impact of enhanced cytokine clearance with sustained high-efficiency daily diafiltration using a mediator-adsorbing membrane (SHEDD-fA) in patients with severe sepsis

**DOI:** 10.1186/cc10990

**Published:** 2012-03-20

**Authors:** O Nishida, T Nakamura, N Kuriyama, K Moriyama, T Miyasho, S Yamada

**Affiliations:** 1 Fujita Health University School of Medicine, Toyoake, Japan; 2 Rakuno Gakuen University, Ebetsu, Japan; 3 Shino-Test Corporation, Sagamihara, Japan

## Introduction

SHEDD-fA is an effective modality that makes the best use of three principles in the treatment of severe sepsis: diffusion, convection and adsorption. We reported the efficacy of SHEDD-fA for the treatment of severe sepsis at the 31st ISICEM 2011 [1]. Here we present the blood clearance (CL) of seven important cytokines with SHEDD-fA.

## Methods

Ten critically ill patients were studied who were on SHEDD-fA, at QB = 150 ml/minute, QF = 1,500 ml/hour (post dilution) and QD = 300 to 500 ml/minute as a nonrenal indication. In order to maximize cytokine adsorption efficiency, we used a large-size (2.1 m^2^) PMMA dialyzer. Blood samples were taken to measure the CL of plasma cytokines (HMGB-1, IL-6, IL-8, IL-10, G-CSF, MCP-1 and MIP-1) at 1 hour and 3 hours after initiation (in one cytokine by 62 to 107 samples).

## Results

**Figure 1:**
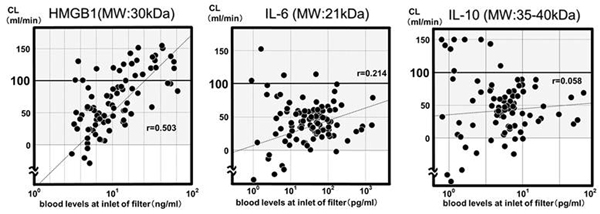
**Correlation between clearance and blood level of cytokines**.

The median values of CL with interquartile ranges of each cytokine (molecular weight: kDa) were: HMGB1 (30 kDa), 53.1 ml/minute (2.1 to 12.5); IL-6 (21 kDa), 39.9 ml/minute (12.4 to 70.6); IL-8 (8 kDa), 64.1 ml/minute (-0.5 to 82.0); IL-10 ml/minute (35 to 40 kDa), 45.6 ml/minute (0.5 to 88.3); G-CSF (19 kDa), 33.2 ml/minute (9.3 to 60.8); MCP-1 (8.7 kDa), 68.5 ml/minute (-14.4 to 125.4); and MIP-1 (7.8 kDa), 66.5 ml/minute (18.6 to 100.0). In particular, CL of HMGB1 was positively correlated with pre-SHEDD-fA blood levels, indicating the mechanism of HMGB1 removal was through adsorption. As a result of enhancing the intensity of the dosage, CL (53 ml/minute) of HMGB1 was higher than that (25 ml/minute) of an *in vitro *experiment that we reported at the 31st ISICEM 2011. See Figure 1.

## Conclusion

Taking into account the fact that the creatinine CL of native kidney function is 100 ml/minute, our findings suggest that SHEDD-fA is a feasible adjusted modality for the treatment of patients with severe sepsis, with or without acute kidney injury. Considering our other laboratory findings, deep filtration may enhance blood clearance.
